# Rating the Intensity of a Laser Stimulus, but Not Attending to Changes in Its Location or Intensity Modulates the Laser-Evoked Cortical Activity

**DOI:** 10.3389/fnhum.2020.00120

**Published:** 2020-03-31

**Authors:** Diana M. E. Torta, Marco Ninghetto, Raffaella Ricci, Valéry Legrain

**Affiliations:** ^1^Institute of Neuroscience, Université catholique de Louvain, Brussels, Belgium; ^2^Health Psychology Research Group, University of Leuven, Leuven, Belgium; ^3^Department of Psychology, University of Turin, Turin, Italy; ^4^Neuroplasticity Laboratory, Nencki Institute for Experimental Biology, Polish Academy of Science, Warsaw, Poland; ^5^Psychological Sciences Research Institute, Université catholique de Louvain, Louvain-la-Neuve, Belgium

**Keywords:** EEG, pain, laser evoked potentials (LEPs), top-down modulation, attention, ratings

## Abstract

Top-down attention towards nociceptive stimuli can be modulated by asking participants to pay attention to specific features of a stimulus, or to provide a rating about its intensity/unpleasantness. Whether and how these different top-down processes may lead to different modulations of the cortical response to nociceptive stimuli remains an open question. We recorded electroencephalographic (EEG) responses to brief nociceptive laser stimuli in 24 healthy participants while they performed a task in which they had to compare two subsequent stimuli on their Spatial location (Location task) or Intensity (Intensity Task). In two additional blocks (Location + Ratings, and Intensity + Ratings) participants had to further provide a rating of the perceived intensity of the stimulus. Such a design allowed us to investigate whether focusing on spatial or intensity features of a nociceptive stimulus and rating its intensity would exert different effects on the EEG responses. We did not find statistical evidence for an effect on the signal while participants were focusing on different features of the signal. We only observed a significant cluster difference in frontoparietal leads at approximately 300–500 ms post-stimulus between the magnitude of the signal in the Intensity and Intensity + Rating conditions, with a less negative response in the Intensity + Rating condition in frontal electrodes, and a less positive amplitude in parietal leads. We speculatively propose that activity in those electrodes and time window reflects magnitude estimation processes. Moreover, the smaller frontal amplitude in the Intensity + Rating condition can be explained by greater working memory engagement known to reduce the magnitude of the EEG signal. We conclude that different top-down attentional processes modulate responses to nociceptive laser stimuli at different electrodes and time windows depending on the underlying processes that are engaged.

## Introduction

Attention can increase or decrease the magnitude of the cortical responses elicited by nociceptive stimuli depending on the processes that are involved (for reviews see Legrain et al., 2012; Torta et al., 2017). Top-down attention towards nociceptive stimuli can be incremented by asking participants to pay attention to specific features of a stimulus, or to provide a rating about its intensity and/or its unpleasantness. Whether these different top-down processes lead to different modulations of the cortical response to nociceptive stimuli remains an open question. In a previous study, Schlereth et al. (2003) asked participants to perform either an intensity or a location discrimination task, while recording the amplitude of the cortical potentials elicited by applying a laser heat stimulus (laser-evoked potentials, LEPs). The authors did not observe significant differences between the location and intensity tasks on the strength of the activity of the source of the LEPs estimated in the bilateral operculo insular cortices, the cingulate gyrus and the contralateral postcentral gyrus in the time window of the N2 and P2, the two main LEP components. In a more recent functional magnetic resonance imaging (fMRI) experiment, Lobanov et al. (2013) instructed participants to selectively attend to changes in either the spatial location or the intensity of two subsequent nociceptive stimuli. By contrasting the cortical activity during the two tasks, they showed that areas of the right posterior parietal cortex exhibited stronger and more sustained activity during the condition wherein participants were tracking spatial changes. Attention to both spatial and intensity features was associated with the activation of frontoparietal regions and the primary somatosensory cortex (S1), with a greater activation of the left dorsolateral prefrontal cortex (DLPFC) in the intensity discrimination task. One possible explanation for the discrepancy in the findings of the two studies is that the sources of the N2-P2 components of the LEPs mainly reflect activity unrelated to the specific processing of features of the stimuli such as their location or intensity. Also, the analysis of the time window of the N2-P2 components of the LEPs might have been too restrictive to identify any difference between conditions, as it implies that differences in the processing of the two features appear between 200–400 ms post-stimulus. Therefore, the first aim of this study was to investigate whether focusing on changes in the location vs. the intensity of the laser stimuli could modulate cortical activity in other post-stimulus intervals.

Previous studies have reported that rating the intensity of a laser or electrical somatosensory stimulus modulates electroencephalographic (EEG) brain activity (Becker et al., 2000; Kanda et al., 2002, see Schoedel et al., 2008 for similar findings obtained using fMRI). More in detail, it was observed that when participants rated the stimuli, an additional late positive component 350–600 ms post-stimulus appeared (Becker et al., 2000; Kanda et al., 2002). This component could not be observed in two control conditions wherein participants did not have to provide any ratings. However, no formal statistical comparison was carried out to confirm the effects, nor was it replicated in further studies. The second aim of the study was to address statistically the question of whether being involved in a rating task during laser stimulation increased the magnitude of the LEPs as compared to conditions during which no rating was required.

Finally, the combined effects of providing a rating on the intensity of the stimulus while performing simultaneously a task on its location are unknown. Previous studies have shown that the amplitude of the LEPs is reduced when participants are engaged in a non-pain related working memory task (Legrain et al., 2013). This would suggest that non-pain related working memory load reduces cognitive resources that would be allocated to the elaboration of the nociceptive stimulus. What happens when the cognitive tasks are both pain-related and have to be shared between different features of the nociceptive stimuli remains elusive. It can be hypothesized that if the discrimination of the spatial features of the stimulus and the discrimination of its intensity require distinct and additional attentional resources, a signal of smaller amplitude in the conditions in which a rating of intensity is requested while performing a spatial task should be expected (i.e., Location + Ratings < Intensity + Ratings and/or Intensity and/or Location). On the other hand, providing a rating of intensity while discriminating the intensity of the stimulus may create a competing situation, as the two operations would share cognitive resources, i.e., discrimination of intensity. In this case, it would be expected that the amplitude of the LEPs is reduced when ratings are provided while participants have to discriminate intensity changes, but not when they have to provide a rating while they attend to the spatial features of the stimulus (e.g., Intensity + Ratings < Intensity and/or Location and/or Location + Ratings). Testing these two hypotheses was the third and final aim of this study.

## Materials and Methods

### Participants

Twenty-four participants took part in this study (11 women, mean age 28.75 ± 4.29, one left-handed). They were recruited among staff members and students of the Université catholique de Louvain and were naïve to the aims of the study. Participants with on going pain, history of chronic pain or neurological diseases were excluded. Before the beginning of the experiment, participants obtained information about the study and signed a written informed agreement to participate. The protocol received ethical approval from the local Ethics Committee in agreement with the convention of Helsinki.

### Stimuli

Nociceptive stimuli were radiant heat stimuli applied to participants’ right-hand dorsum using an Nd:YAP laser (wavelength 1.34 μm; Stimul 1340 El.En. Firenze, Italy). The stimulus duration was 5 ms, and the laser beam diameter at the target site was 5 mm. Stimulation intensity (in Joules) was adjusted individually before the beginning of the experiment to elicit a clear pinprick sensation and a reaction time smaller than 650 ms compatible with the activation and the conduction velocity of Aδ-fibers (Towell et al., 1996; Mouraux et al., 2003; Churyukanov et al., 2012). Two stimulation intensities were defined individually based on the participant’s perception using a numerical rating scale (NRS) ranging from 0 to 100, where 0 referred to *no perception* and 100 to *as intense as this stimulus could be*. High-intensity stimuli were set at an energy eliciting a percept rated around 60–70, medium intensity stimuli were set at an energy eliciting a percept rated around 40–50. The resulting intensities were of 4.03 ± 0.52 J for the medium intensity and an average of 4.27 ± 0.52 J for the high-intensity stimuli. During the experiment, the direct vision of the participant’s hand, of the laser probe, and the experimenter was prevented by employing a wooden screen.

### Procedure

Participants were seated in a dimly lit room with their right hand positioned on a table. The experiment consisted of four experimental blocks, one per condition, presented according to a counterbalanced and pre-defined order across participants. The predefined sequence prevented participants to receive more than three consecutive stimuli having the same intensity or location. Each block was composed of 20 stimuli, 10 high-intensity and 10 medium-intensity stimuli delivered in pseudorandom order. Ten stimuli were applied on the medial part on the right hand, and 10 on the lateral part, the two parts being dissociated by the third metacarpal bone. Stimuli were triggered manually by the experimenter using an inter-stimulus time interval ranging from 8 to12 s. A small fixation point was positioned on the wooden screen.

During the *Location* (L) condition, participants were requested to selectively attend to changes in stimulus location and were asked to report at each trial if the stimulus was applied on the same vs. a different location as the preceding one. During the *Intensity* (I) condition, participants were requested to selectively attend to changes in stimulus intensity and were asked to report at each trial if the stimulus was applied with the same or a different intensity as the preceding one. Importantly, changes in intensity and location both occurred within the same block of stimulation, but participants were requested to focus only on changes in one of the two features. In this way, we were able to control for the relative contribution of a change in the characteristics of the stimulus and isolate the effects of feature-related selective attention (see also Lobanov et al., 2013). In the other two conditions, participants were requested to perform one of the two tasks and additionally provide, using the NRS, a rating of the intensity of each stimulus after having reported the change in location [block *Location + Rating* (LR)] or intensity [block *Intensity + Rating* (IR)].

Responses were reported verbally. Participants were encouraged to answer as accurately as possible, but they did not receive any specific instruction regarding speed. The accuracy of the task and the ratings were recorded.

**Figure 1:**
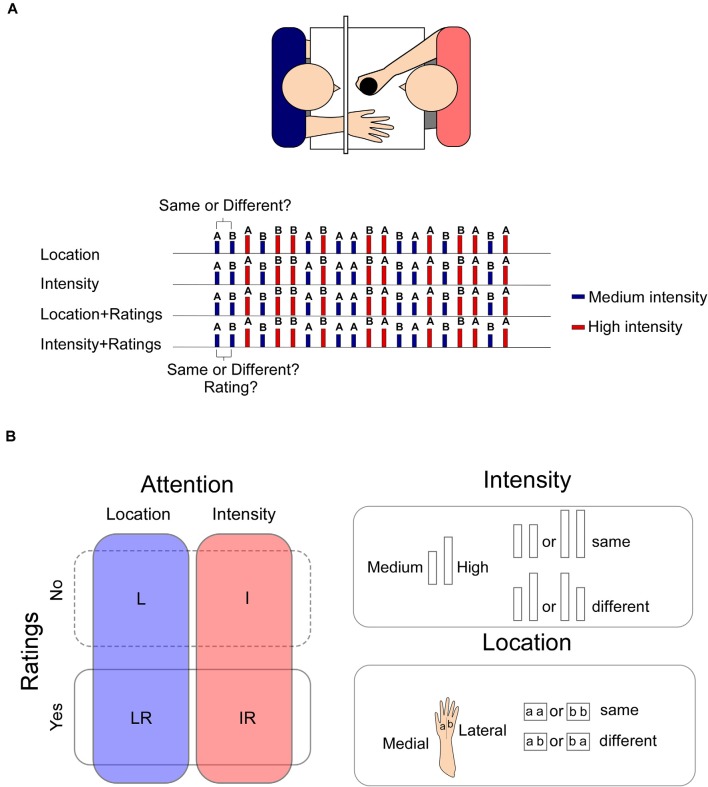
Panel **(A)** upper panel: the stimuli were applied manually on the participant’s right hand by one of the experimenters who was completely covered from view. The second experimenter collected the ratings and the responses. Lower panel: an example of the sequence of the stimuli. In LR and IR blocks, the response to the task was collected before the rating. Panel **(B)** left panel: the experiment was composed of four blocks in which participants had to focus either on changes in the location or on the intensity of the stimulus (blocks L and I). In two additional blocks, participants had also to provide a rating of the intensity of the stimulus (LR, IR). Stimuli could be of a High or a Medium intensity. Right panel: two intensities were used “High” or “Medium,” which were selected at the beginning of the experiment to elicit respectively a percept of 60/70 or 40/50 out of 100. Two consecutive high intensity or low intensity stimuli were considered “same.” Two consecutive stimuli of different intensities were considered “different” irrespective of the direction of the change (high-medium or medium-high). Two stimuli applied onto the same hand sector, whether medial or later were considered “same,” whereas two consecutive stimuli applied onto the later or medial sector of the hand were considered “different” irrespective of the direction of the change (lateral-medial or medial-lateral).

See Figure 1 for details of the experimental procedure.

### Behavioral Measures

For each participant, accuracy was measured as the proportion of correct answers in each of the four conditions. Ratings of perceived intensity of the stimuli were averaged separately for the LR and IR blocks.

### Electroencephalogram

The electroencephalogram (EEG) was recorded at a 1 kHz sampling rate using a 64-channel amplifier and digitizer (ASA-LAB EEG system; Advanced Neuro Technologies, The Netherlands). Scalp signals were acquired with an average reference, using 64 shielded Ag-AgCl electrodes, positioned according to the 10–10 system (Waveguard; Advanced Neuro Technologies, The Netherlands). The ground electrode was positioned at FCz. Analysis of the EEG data was carried out using Letswave 6^1^.

The continuous average-referenced EEG recordings were first band-pass filtered using 0.3–30 Hz Butterworth zero-phase (4th order filter) and then segmented in 2-s epochs extending from −0.5 to +1.5 s relative to stimulus onset. EOG artifacts were subtracted using independent component analysis (ICA; Jung et al., 2000). In all datasets, ICs related to eye movements had a large EOG channel contribution and a frontal scalp distribution. Baseline correction was performed by subtracting the −0.5 to 0 s pre-stimulus interval. Epochs exceeding ±100 μV were excluded. Artifact-free epochs were finally averaged for each condition (L, I, LR, IR) and each participant.

### Statistical Analysis

The proportion of correct answers was analyzed using a two-way repeated-measure ANOVA with *Task* (Location vs. Intensity) and *Ratings* (presence vs. absence) as within-participant factors. Perceived intensity in the LR and IR conditions was compared with a *t*-test for paired measures. The significance level was set at *p* ≤ 0.05. Effect sizes were measured using partial Eta squared for the ANOVA.

#### Cluster-Based Permutation Test

To explore whole scalp EEG brain activity concomitantly correcting for multiple comparisons, we performed a non-parametric temporal cluster-based permutation test on the entire duration of the epoch (−0.5 to 1.5 s). The cluster-based permutation test allows for resolving the issue of multiple comparisons of point-by-point analysis (Maris and Oostenveld, 2007; Maris, 2012). Two thousand permutations were used per comparison (L vs. LR, I vs. IR, L vs. I, LR vs. IR) to obtain a reference distribution of maximum cluster magnitude. Finally, the proportion of random partitions that resulted in a larger cluster-level statistic than the observed one (i.e., *p*-value) was calculated. Clusters in the observed data were regarded as significant if they had a magnitude exceeding the threshold of the 95th percentile of the permutation distribution (corresponding to a critical alpha-level of 0.05; see also van den Broeke et al., 2015, 2016). The critical alpha-level was lowered to 0.012 to account for the four comparisons. Nevertheless, the threshold for electrodes was deliberately left less stringent due to the exploratory nature of the study and the power characteristics of the permutation test as compared to other methods of false discovery rate (Lage-Castellanos et al., 2010).

## Results

### Behavior

#### Behavioral Measures

**Figure 2:**
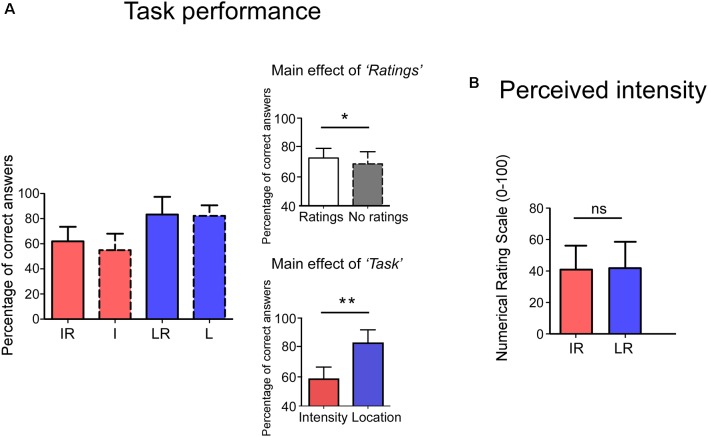
Panel **(A)**, the y-axis shows the percentage of correct answers. Accuracy was higher in the Rating conditions (Main effect of Rating) and in the Location Task (Main effect of Task). Interactions were not significant. Panel **(B)**, no differences were observed in the perceived intensity for the two tasks. The y-axis represents the perceived intensity on a Numerical Rating Scale (NRS) ranging from 0 to 100. IR = Intensity + Ratings, I = Intensity LR = Location + Ratings, L = Location. *p < 0.05, **p < 0.001, ns, not significant.

Behavioral results are summarized in Figure 2. The participants’ proportion of correct answers was significantly higher in the *Location* conditions than in the *Intensity* ones (Main effect of *Task*: *F*_(1,23)_ = 91.04 *p* < 0.001, $\eta_{\text{p}}^{2}$ = 0.80). Moreover, the proportion of correct answers was higher when a rating was present than when it was absent (Main effect of *Rating*: *F*_(1,23)_ = 5.35 *p* = 0.030, $\eta_{\text{p}}^{2}$ = 0.19). The interaction between the two factors was not significant (*F*_(1,23)_ = 1.10 *p* = 0.304, $\eta_{\text{p}}^{2}$ = 0.05). No significant difference was observed for the perceived intensity (*t*_(23)_ = −0.664 *p* = 0.513) between the IR and LR conditions.

### EEG

**Figure 3:**
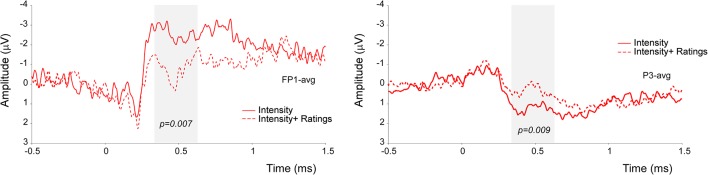
Results of the non-parametric cluster-based permutation test in the time-domain. The signal in the Intensity condition was significantly more negative at the FP1 electrode, and significantly more positive at the P3 electrode.

Significant amplitude differences were observed between the I and IR conditions at a latency later than 300 ms after stimulus onset. However, only differences with a *p*-value smaller than 0.012 (see the statistical paragraph) were considered significant. All findings are summarized in Table 1, including those which resulted significant at the cluster-based permutation test, but did not survive the 0.012 cut. More specifically, the signal was more negative in frontal electrodes and more positive in parietal electrodes in the *Intensity* condition (see Figure 3).

**Table 1:** Results of the cluster-based permutation test.

Electrode	Time window	*p*-value
FP1	0.34–0.54	0.007
CP5	0.80–0.90	0.039
CP1	0.40–0.54	0.028
P3	0.36–0.54	0.009
AF7	0.35–0.50	0.026
CPz	0.4–0.55	0.021
P1	0.36–0.49	0.039

*Electrodes in red highlight differences still significant after setting the α cut off at 0.012. All electrodes showed differences at the *p* < 0.05 level*.

We did not find any significant difference when comparing the signal of the L vs. LR, the IR vs. LR, and the L vs. I conditions.

#### Control Analyses

Considering that we observed a significantly better performance at the *Location* task that might have been indicative of the difficulty of the task, we investigated whether this affected the amplitude of the N2 and P2. We reasoned that a greater difficulty of the task should be associated with greater cognitive load, which in turn has been shown to reduce the amplitude of the LEP signal (Legrain et al., 2013), at least when the task was non-pain related.

**Figure 4:**
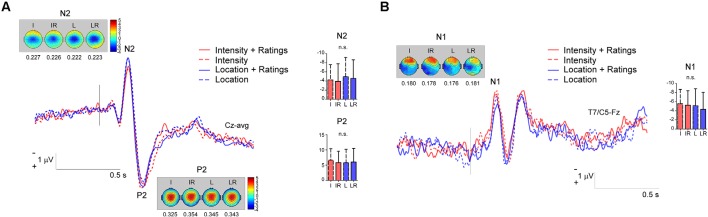
Panel **(A)** shows the magnitude of the N2 and P2 components in the four conditions; panel **(B)** the magnitude of the N1 potential. n.s., non-significant.

To carry out such analysis we used the same pre-processed signal that was used for the cluster based permutation test. Individual values of the N1, N2 and P2 were extracted for each participant. The N2 was defined as the most negative deflection at Cz in the 170–250 ms interval, the P2 as the most positive deflection at Cz following the N2. The N1 was extracted by first re-referencing to Fz the averaged signal, and then extracting the average of the peaks at T7/C5 in the 120–220 ms interval. The data were then analyzed by using a two-way repeated measure ANOVA with the factors *Task* and *Rating*. The magnitude of the N1, N2 and P2 was not modulated by the tasks, indeed all *p*-values were >0.05 (see Figure 4; N2: Main effect of *Task* *F*_(1,23)_ = 2.192, *p* = 0.152 $\eta_{\text{p}}^{2}$ = 0.084, Main effect of *Rating* *F*_(1,23)_ = 0.533 *p* = 0.472 $\eta_{\text{p}}^{2}$= 0.022, Interaction *Task* × *Rating* *F*_(1,23)_ = 0.022 *p* = 0.883 *η*^2^ = 0.001; P2: Main effect of *Task* *F*_(1,23)_ = 0.535, *p* = 0.472 $\eta_{\text{p}}^{2}$ = 0.023, Main effect of *Rating* *F*_(1,23)_ = 0.394 *p* = 0.536 *η*^2^ = 0.017, Interaction *Task* × *Rating* *F*_(1,23)_ = 2.298 *p* = 0.143 $\eta_{\text{p}}^{2}$= 0.091; N1: Main effect of “Task” *F*_(1,23)_ = 3.290, *p* = 0.083 $\eta_{\text{p}}^{2}$= 0.125, Main effect of “Rating” *F*_(1,23)_ = 1.688 *p* = 0.207 $\eta_{\text{p}}^{2}$ = 0.068, Interaction “Task” × “Rating” *F*_(1,23)_ = 0.170 *p* = 0.684 $\eta_{\text{p}}^{2}$ = 0.007).

**Figure 5:**
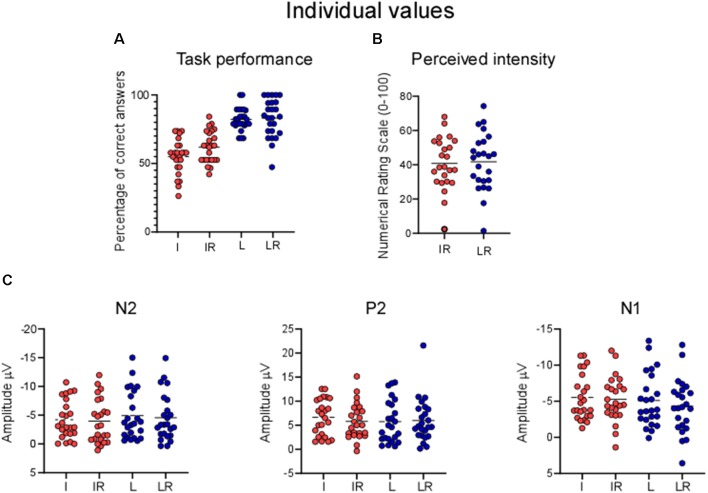
Individual values for **(A)** percentage of correct answers, **(B)** perceived intensity, **(C)** LEPs. Each point represents one participant, horizontal lines are means.

Figure 5 shows the distribution of the individual values.

## Discussion

This study was designed to investigate whether different top-down attentional processes led to different modulations of the cortical response to nociceptive stimuli. More specifically, we assessed whether: (i) performing a task during which participants focused on changes either in the location or in the intensity of the laser stimuli could modulate the magnitude of the LEP responses in a time window broader than that of the N2 and P2 peaks; (ii) providing a rating of the intensity could modulate the magnitude of the brain responses; and (iii) discriminating the location or intensity of a stimulus while providing a rating of its intensity could influence the amplitude of the LEPs.

Our results provide no statistical evidence indicating that focusing on either the location or the intensity of the laser stimuli would modulate the magnitude of the induced cortical responses, not even in time intervals extending beyond that of the N2-P2 complex.

Also, our findings did not disclose any statistical significance regarding the competing effect of focusing on the *location* of the stimulus while providing a rating of its intensity. However, our data indicate that a difference between the conditions in the window approximately ranging from 340 to 540 ms post-stimulus. More specifically, we observed that the signal was more negative at anterior leads (FP1) and more positive in posterior ones (P3) when no rating had to be provided.

### Tracking Changes Between Location and Intensity: Behavioral and EEG Differences

Our results highlight a significantly better behavioral performance at the *Location* tasks, as compared to the *Intensity* ones. This is most likely due to the nature of the task: the two locations to be discriminated were distinguishable, being the lateral or medial side of the hand. So even if the laser beam was displaced after each stimulus, the relative distance between two consecutive stimuli applied at a *different* location (i.e., in the lateral or medial portions of the hand) was clearly above the threshold of a minimally detectable change (Frahm et al., 2018). In contrast, distinguishing the intensity of two consecutive stimuli was more challenging, due to the intrinsic variance of the perception of different laser heat stimuli. Please also note that in contrast with other studies (Schlereth et al., 2003; Mancini et al., 2016), we did not choose parameters of intensity of location that would match the participants’ performance in the two tasks, nor did we provide feedback about the quality of their performance (Mancini et al., 2016). One might argue that such a methodological difference might have reduced the possibility of observing EEG differences in the *Location* and *Intensity* blocks. Indeed, these effects could have been masked by the effects of a different cognitive load, *per se* exerting a modulation on the amplitude of the signal. While we cannot rule out this possibility, we find it is unlikely the only explanation for our findings. First, the cognitive load requested by the two tasks was not sufficiently different to affect the magnitude of the signal for the main components (see Figures 4, 5). Second, Schlereth et al. (2003) matched the two tasks for difficulty but observed the same findings as our present study. We, therefore, conclude that the possibility of observing differences in the modulation of cortical activity to heat stimuli due to *Intensity* or *Location* tasks strongly depends on the methodological approach that is used.

These results also challenge the possibility that the increase observed in the magnitude of the LEPs in the 100–200 ms post-stimulus, i.e., during the time windows of the negativities of the LEPs (for a review, see Legrain et al., 2002, 2012) depends on focusing on the spatial characteristics of the stimulus. Rather, we conclude that spatial attention leads to an increase in the signal only when its effects are contrasted with conditions requiring displacement of attention away from the stimulated body location (e.g., attention allocated to the other hand). *Per se* we found no statistical evidence supporting the possibility that paying attention to spatial features of a stimulus affects the signal, neither in the N2 P2 nor in later time windows.

### Effects of Rating the Stimuli

Our behavioral data also highlighted a significant, although smaller, effect of providing a rating in the accuracy of the performance. More specifically, providing a rating of intensity improved the percentage of correct answers of the discrimination tasks, irrespective of the condition (Location or Intensity). A possible interpretation is that providing the rating resulted in a greater attentional overall engagement in the task.

In line with previous reports (Becker et al., 2000; Kanda et al., 2002; van den Broeke et al., 2016, 2019), our EEG findings show that when participants were asked to provide ratings about a laser stimulus, a more positive wave appeared in parietal electrodes, at the same time window as the one observed in the present study. Therefore, we can speculate that responses occurring approximately 340–540 ms after the onset of the laser stimulus are influenced by the decisional process related to reporting the subjective perception of the stimulus. However, contrary to our present results, previous studies found an increase rather than a decrease of the amplitude in the conditions in which ratings had to be provided. We propose that this may be due to the nature of the task. Indeed, neither in Kanda et al. (2002), nor Becker et al. (2000) were participants performing a discrimination task on the intensity characteristics of the stimulus, having to additionally provide a rating of its intensity.

### Intensity vs. Intensity + Ratings

Our data also did not provide statistical evidence for significant effects of performing a spatial discrimination task while rating the intensity of the stimulus. We reasoned that focusing on the spatial location of a stimulus while providing a rating of its intensity could have an impact on the amplitude of the signal, as the two tasks might have been tapping on different cognitive resources (Lobanov et al., 2013). Alternatively that providing a rating of intensity while performing a discriminative task would result in a greater cognitive load due to competing resources. Our results support the second possibility.

Notably, the differences in the LEP magnitude were most prominent at the electrodes located on the left hemisphere (Fp1 and P3). This topographical lateralization is similar to the results obtained in fMRI by Lobanov et al., 2013 showing engagement of the left DLPFC in the discrimination of Intensity changes.

Neuroimaging and transcranial magnetic stimulation (TMS) human data and single-unit monkey recordings have consistently implicated a parietal-prefrontal network in the magnitude estimation of time, size, space, and numbers (Walsh, 2003; Lewis and Miall, 2006; Bueti and Walsh, 2009). Although a dominance of the right hemisphere has been proposed for the magnitude evaluation of numbers and space, it remains to be elucidated whether the left hemisphere becomes dominant in tasks involving motor selection (see a discussion in Bueti and Walsh, 2009). The fact that nociceptive stimuli have high behavioral relevance and can trigger motor responses to prompt defensive actions (Legrain et al., 2009; Moayedi et al., 2015; Algoet et al., 2018) can explain the lateralization of the network observed in the present data.

Our findings also highlight a greater magnitude of the signal in the *Intensity* rather than the *Intensity + Rating* condition at Fp1. Previous findings have involved the same prefrontal-parietal network in working memory (Lewis and Miall, 2006) and past observations have suggested that LEPs responses are reduced when LEPs are delivered during the execution of a non-pain related working memory task (Legrain et al., 2013). Therefore we speculate that the smaller amplitude of the signal in the *Intensity + Rating* condition may be explained by the greater working memory engagement necessary to provide the rating. We can only hypothesize why the differences between the rating and non-rating conditions did not emerge for the *Location* task. One possibility is that the greater difficulty of the *Intensity* task prompted a greater attentional engagement and therefore boosted the task effects.

To conclude, our results do not provide statistical support to the possibility that paying attention to spatial or intensity features of a stimulus affects the amplitude of EEG signals after the administration of a laser stimulus. Nevertheless, we observed that providing a rating of the intensity of a stimulus while discriminating its intensity may lead to a smaller signal. Overall, our findings also promote awareness of the importance of the control conditions in the context of top-down attentional manipulation of nociceptive stimuli.

## Data Availability Statement

The datasets generated for this study are available on request to the corresponding author.

## Ethics Statement

The studies involving human participants were reviewed and approved by UC Louvain ethical commission. The participants provided their written informed consent to participate in this study.

## Author Contributions

DT designed research and performed data analysis. DT and MN performed data collection. All authors critically discussed the results, reviewed the article and gave their final approval.

## Conflict of Interest

The authors declare that the research was conducted in the absence of any commercial or financial relationships that could be construed as a potential conflict of interest.

## Footnotes

^1^https://www.letswave.org/
